# Multielemental Composition of Suet Oil Based on Quantification by Ultrawave/ICP-MS Coupled with Chemometric Analysis

**DOI:** 10.3390/molecules19044452

**Published:** 2014-04-10

**Authors:** Jun Jiang, Liang Feng, Jie Li, E Sun, Shu-Min Ding, Xiao-Bin Jia

**Affiliations:** 1 Affiliated Hospital on Integration of Chinese and Western Medicine, Nanjing University of Chinese Medicine, Xianlin Avenue 138#, Xianlin University City, Nanjing 210023, Jiangsu, China; E-Mails: xuyan9323@126.com (J.J.); wenmoxiushi@126.com (L.F.); lijiejstcm@163.com (J.L.); sune0825@163.com (E.S.); dsm11@163.com (S.-M.D.); 2 Key Laboratory of New Drug Delivery System of Chinese Meteria Medica, Jiangsu Provincial Academy of Chinese Medicine, 100# Shizi Road, Nanjing 210028, Jiangsu, China

**Keywords:** suet oil (SO), multielements, ultrawave digestion, ICP-MS, chemometrics analysis

## Abstract

Suet oil (SO) has been used commonly for food and medicine preparation. The determination of its elemental composition has became an important challenge for human safety and health owing to its possible contents of heavy metals or other elements. In this study, ultrawave single reaction chamber microwave digestion (Ultrawave) and inductively coupled plasma-mass spectrometry (ICP-MS) analysis was performed to determine 14 elements (Pb, As, Hg, Cd, Fe, Cu, Mn, Ti, Ni, V, Sr, Na, Ka and Ca) in SO samples. Furthermore, the multielemental content of 18 SO samples, which represented three different sources in China: Qinghai, Anhui and Jiangsu, were evaluated and compared. The optimal ultrawave digestion conditions, namely, the optimal time (35 min), temperature (210 °C) and pressure (90 bar), were screened by Box-Behnken design (BBD). Eighteen samples were successfully classified into three groups by principal component analysis (PCA) according to the contents of 14 elements. The results showed that all SO samples were rich in elements, but with significant differences corresponding to different origins. The outliers and majority of SO could be discriminated by PCA according to the multielemental content profile. The results highlighted that the element distribution was associated with the origins of SO samples. The proposed ultrawave digestion system was quite efficient and convenient, which could be mainly attributed to its high pressure and special high-throughput for the sample digestion procedure. Our established method could be useful for the quality control and standardization of elements in SO samples and products.

## 1. Introduction

Inorganic elements, important chemical components of natural foods and their products, have been found to play an important role in human safety and health. Based on emerging evidence heavy metals and metalloids, especially Pb, As, Hg and Cd, have attracted increasing attention due to their bioavailability and toxicity. These elements may be introduced into the food chain in various ways, including contamination during cultivation, processing, and storage. Other elements such as Mg, Ca, Mn, Ni, Cu, Zn, and Fe are also present and found to have both nutritional and toxic effects for human health [1,2,3].

Suet oil (SO), obtained from *Capra hircus linnaeus* or *Ovis aries linnaeus*, contains abundant inorganic elements [4]. SO can be widely applied in the food industry, health protection industry and various fields of medicine [5,6,7]. For example, SO can be used for the processing of the traditional herb Herba Epimedii. It can be assembled into smaller size nanomicelles and then promotes the absorption and enhances the clinical efficacy of the main active flavonoids [1,8,9,10]. However, inorganic elements can significantly reduce the critical micelle concentration (CMC) of the surfactant (SO) which inhibits the formation of micelles [11,12,13,14,15]. In addition, the inorganic elements in SO can have nutritional functions or toxic effects for human health. Therefore, the quality control of inorganic elements in SO can ensure its safety and function, and also provide important data for the use of SO in the food and medicine industries.

Inductively coupled plasma mass spectrometry (ICP-MS) is a powerful technique for elemental analysis in various fields [16,17]. Before ICP-MS instrument analysis, sample digestion efficiency is a critical pretreatment step affecting analytical results, especially for oleaginous matrices [18], such as SO. Microwave-assisted digestion including closed vessel systems has been widely used as an original way to pretreat samples for ICP-MS analysis [19,20]. The ultrawave single reaction chamber microwave digestion system overcame the limitations of traditional microwave sample preparation. At the heart of this system, there is a large sample chamber which is pre-pressurized with an inert gas and heated with microwave energy. The chamber serves both as a microwave cavity and a reaction vessel. Different samples can be digested simultaneously because no vessel assembly/disassembly is required while vessel cleaning is eliminated with the use of disposable glass vials. Direct temperature and pressure control of every sample ensures complete control of the digestion process. Blanks are significantly lower than with closed vessel digestion, since less solution transfer occurs, quartz vials can be used and digestion acid volumes are lower. 

Although it has been used widely in the food and medicine industries, to the best of our knowledge, the standardization of SO still remains a challenge due to contamination or purposeful adulteration with other oils. It is difficult to identify the origins and species of SO based on their appearance and morphology. In this study, eighteen batches of SO were collected from three origins. Ultrawave digestion parameters (digestion time, digestion temperature, digestion pressure) were optimized by Box-Behnken Design (BBD) statistical screening and applied to the pretreatment of SO for the subsequent determination of Pb, As, Hg, Cd, Fe, Cu, Mn, Ti, Ni, V, Sr, Na, Ka and Ca by ICP-MS. Finally, principal component analysis (PCA) was performed to evaluate and classify the eighteen batches of SO according to the detected contents of the various elements.

## 2. Results and Discussion

### 2.1. Optimization for SO Ultrawave Digestion by Box-Behnken Design

Ultrawave digestion can be used to save consumables costs and sample pretreatment time owing to its higher performance and throughput. In addition, ultrawave digestion could be operated up to 199 bar pressure and 240 °C temperature, which can easily digest oleaginous matrices. Box-Behnken design (BBD), a collection of mathematical and statistical techniques, was first established by Box and Wilson [21]. It has been widely applied for improving or optimizing processing conditions in food and pharmaceutical studies [22].

**Figure 1:**
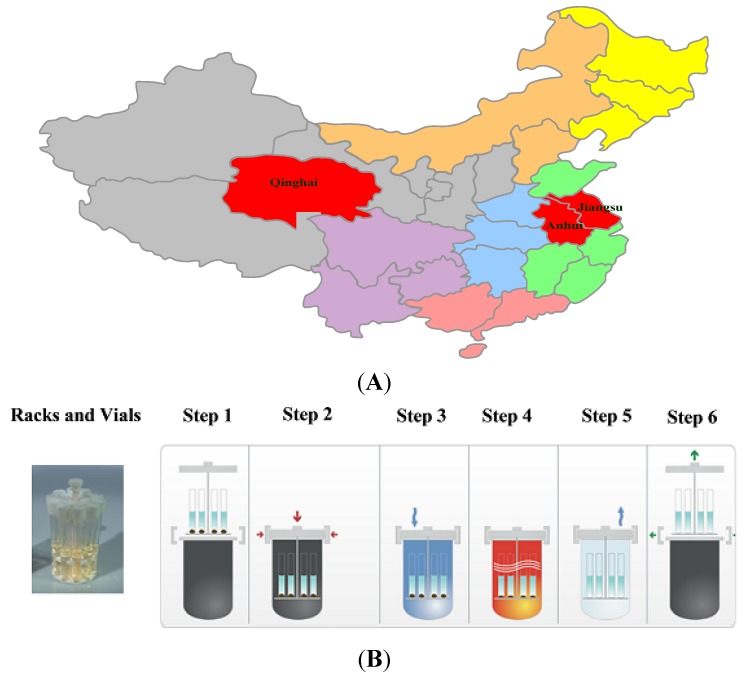
Origins of the samples and the ultrawave digestion procedure. (**A**) The distribution of the 18 batches of SO in China; (**B**) The ultrawave digestion procedure.

In this study, one batch of SO sample obtained from Jiangsu was used to optimize the digestion conditions by BBD (Figure 1), and its blank and spiked samples were pretreated in parallel three times. Then the recovery was calculated as follows:

[(Measured value − Original value)/Spiked value] × 100%.

Finally, the optimal conditions for digestion were screened out by BBD. The ultrawave digestion procedure is shown in Table 1. Operating conditions and parameters for the ICP-MS and ultrawave single reaction chamber microwave digestion systems are shown in Table S1. As shown in Table S2, three digestion variables including temperature (A), time (B) and pressure (C) were examined by BBD design. In this optimization, the recoveries of 14 metal elements were chosen as the response. Comprehensive test results of response surface plots (3D) showed that the recoveries of 14 elements were all higher than 80% under the experimental conditions of temperature at 210 °C, pressure at 90 bar and digestion time for 35 min. 

**Table 1:** Ultrawave digestion procedure.

Steps	Status	Temperature/°C	Time/min
1	1600 W heating	25–120	5
2	Insulation	120	5
3	1600 W heating	120–210	5
4	Insulation	210	35

Precharged nitrogen pressure of 90 bar.

**Figure 2:**
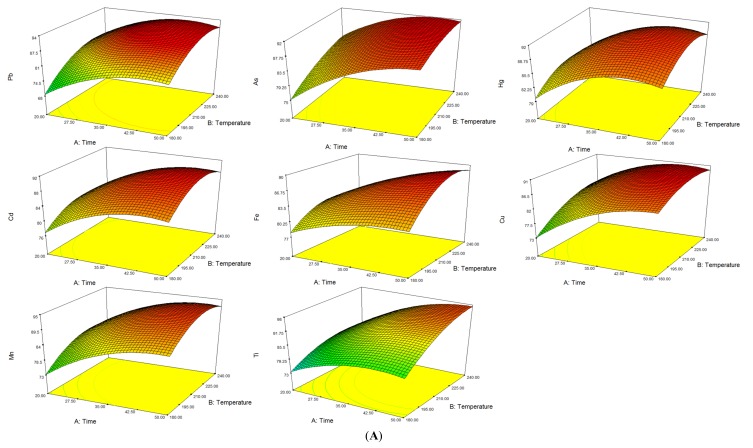

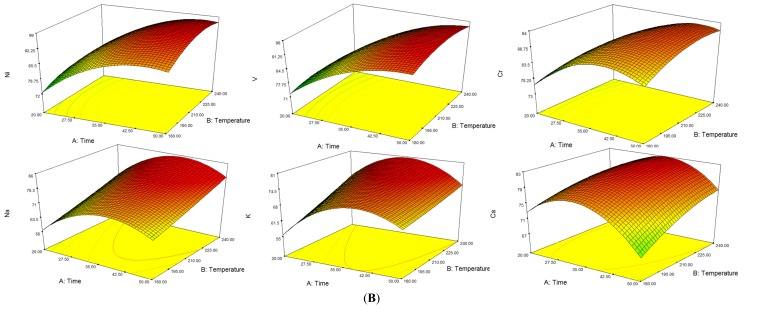
Optimization of ultrawave digestion parameters by BBD. (**A**) Response surface plots (3 D) of Pb, As, Hg, Cd, Fe, Cu, Mn, Ti; (**B**) Response surface plots (3 D) of Ni, V, Cr, Na, K, Ca.

The experimental data was analyzed by ANOVA and the results are given in Table 2 and Table S2. Values of “prob > F” less than 0.001 indicated that the model terms are significant. R-Squared higher than 0.99 for less than 1.0% of the total variations, indicated that the optimal results were accurate and reliable. Therefore, we could find out the optimal condition from (3D) response surface plots was a temperature at 210 °C while the pressure was kept at 90 bar for 35 min (Figure 2). The correlation coefficient of 14 elements were all at 0.99 under ICP-MS conditions, while the standard curves were forced through the origin. Blank sample was repeatedly testing 10 times, and three times the standard deviation was the limit of detection (LOD), and 10 times the standard deviation was the limit of quantitation (LOQ). The LOD were 0.002–0.6 μg/kg, LOQ were 0.335–9.1 μg/kg, the recovery rates of 14 standards varied from 86.5%–99.2% (Table 3), revealing the accuracy of this method.

**Table 2:** ANOVA for response surface model.

Response	Final Equation	Std. Dev.	Mean	C.V. %	PRESS	R-Squared
Pb	Pb = 85.72 + 3.82A + 4.94B + 10.24C − 0.97AB + 2.28AC + 1.05BC − 5.03A^2^ − 4.68B^2^ − 4.79C^2^	6.32	75.81	8.34	2913.15	0.9021
As	As = 83.56 + 4.29A + 2.96B + 11.96C − 1.36AB + 1.69AC − 0.44BC − 3.30A^2^ − 1.20B^2^ − 5.46C^2^	5.57	76.76	7.26	2152.43	0.9035
Hg	Hg = 84.15 + 2.78A + 2.02B + 13.83C + 0.15AB + 1.48AC − 1.62BC − 3.74A^2^ − 2.89B^2^ − 5.56C^2^	4.95	75.81	6.52	1632.57	0.9341
Cd	Cd = 82.92 + 3.13A + 2.37B + 13.21C + 0.30AB + 1.88AC − 1.27BC − 3.40A^2^ − 3.58B^2^ − 5.07C^2^	5.88	74.69	7.87	2239.73	0.9038
Fe	Fe = 81.10 + 1.80A + 1.80B + 11.51C + 1.20AB + 2.23AC + 0.53BC − 1.48A^2^ − 3.25B^2^ − 4.19C^2^	4.58	75.01	6.10	1054.59	0.9175
Cu	Cu = 83.23 + 3.44A + 4.06B + 10.68C − 1.36AB + 2.51AC − 1.36BC − 3.46A^2^ − 2.27B^2^ − 4.25C^2^	4.77	76.42	6.25	955.60	0.9151
Mn	Mn = 85.88 + 5.23A + 4.67B + 10.41C − 0.50AB + 1.60AC − 2.02BC − 4.09A^2^ − 4.20B^2^ − 2.82C^2^	5.40	78.30	6.90	1939.88	0.9031
Ti	Ti = 86.85 + 5.07A + 3.30B + 8.45C + 1.96AB + 1.96AC + 3.11BC − 4.23A^2^ − 3.47B^2^ − 2.97C^2^	3.32	79.56	4.17	253.82	0.9498
Ni	Ni = 89.17 + 7.35A + 3.41B + 9.18C + 0.89AB + 3.39AC − 1.64BC − 6.99A^2^ − 3.68B^2^ − 2.36C^2^	3.92	80.27	4.89	726.98	0.9517
V	V = 87.65 + 7.26A + 2.41B + 9.21C + 0.85AB + 6.05AC − 3.25BC − 5.74A^2^ − 2.96B^2^ − 4.18C^2^	3.23	78.85	4.10	789.74	0.9671
Cr	Cr = 86.95 + 5.05A + 1.37B + 8.23C + 2.34AB + 2.14AC − 0.39BC − 6.88A^2^ − 2.58B^2^ − 3.33C^2^	6.02	78.22	7.70	2293.24	0.9060
Na	Na = 73.30 + 2.44A + 4.97B + 10.24C − 0.075AB + 3.30AC + 2.08BC − 10.32A^2^ + 0.30B^2^ − 4.91C^2^	5.70	63.11	9.04	2112.79	0.9205
K	K = 71.15 + 0.54A + 2.90B + 9.96C − 2.14AB + 3.66AC + 2.99BC − 9.80A^2^ + 0.29B^2^ − 5.65C^2^	6.64	60.80	10.92	3005.07	0.9062
Ca	Ca = 78.15 + 1.02A + 0.82B + 8.36C + 1.79AB − 2.49AC + 2.54BC − 7.43A^2^ − 1.53B^2^ − 4.41C^2^	4.92	69.03	7.12	1449.21	0. 9176

A, Time; B, Temperature; C, Pressure.

**Table 3:** The linear regression equations, the correlation coefficient (r), method detection limits(LOD, LOQ), precision, repeatability, and recovery of 14 elements under ICP-MS conditions.

Elements	Linear equation	Linearity range μg/L	r	LOD μg/kg	LOQ μg/kg	Precision (RSD, n = 6) %		Repeatability (RSD, n = 6) %	Recovery (%)
						Intraday	Interday		
Pb	Y = 66211X	0–10	0.9998	0.005	0.353	1.23	1.56	2.36	97.8
As	Y = 1487.9X	0–5000	1.0000	0.02	2.56	2.31	2.43	2.07	98.6
Hg	Y = 6572.3X	0–10	1.0000	0.004	0.708	1.43	1.63	3.24	96.4
Cd	Y = 3955.5X	0–10	1.0000	0.002	0.335	2.03	2.34	2.78	98.3
Fe	Y = 215.19X	0–5000	0.9981	0.09	3.64	1.78	2.44	2.78	97.4
Cu	Y = 14242 X	0–10	0.9996	0.008	3.091	2.58	1.96	3.35	88.7
Mn	Y = 7966.1X	0–50	1.0000	0.02	5.18	2.32	2.55	2.23	93.8
Ti	Y = 168.39X	0–500	0.9993	0.2	5.8	1.73	2.05	2.19	86.5
Ni	Y = 10693X	0–50	0.9982	0.009	5.668	1.99	2.57	2.47	90.4
V	Y = 9399.3X	0–10	1.0000	0.003	2.608	2.27	2.79	2.94	96.5
Cr	Y = 1441.2X	0–50	1.0000	0.002	6.088	1.66	2.16	2.38	99.2
Na	Y = 45249X	0–5000	0.9988	0.2	9.1	1.07	2.76	3.76	89.7
K	Y = 15646X	0–5000	0.9979	0.6	8.1	2.06	2.37	2.89	90.2
Ca	Y = 51.87X	0–500	0.9975	0.3	5.1	1.65	2.39	3.25	87.9

### 2.2. Application to Multielemental Analysis in SO

In this study, fourteen elements in SO samples were successfully determined to compare each one. Under our experimental conditions, the digested solutions containing of the fourteen elements, including Pb, As, Hg, Cd, Fe, Cu, Mn, Ni, Ti, V, Cr, Na, K and Ca, were directly injected and analyzed after constant volume.

As shown in Table 4, an obvious difference in the concentrations of all 14 elements was observed in different SO samples. The contents of elements detected in various samples were in the range from 0.0015 to 1.4 mg/kg for Pb, from 0.013 to 784 mg/kg for As, from 0.0015 to 0.025 mg/kg for Hg, from 0.239 to 1.61 mg/kg for Cu, from 196 to 817 mg/kg for Na, from 94 to 584 mg/kg for K, from 18 to 80 mg/kg for Ca, from 3.81 to 21 mg/kg for Ti, from 0.079 to 0.274 mg/kg for V, from 4.00 to 9.81 mg/kg for Cr, from 0.61 to 2.03 mg/kg for Mn, and from 71.6 to 224 mg/kg for Fe.

Among the analyzed elements, Na was the most abundant element, followed by K, Fe, Ca, Ti, Cr, Ni, Mn, Cu, V, As, Pb, Hg, and Cd. In particular, the content of elements such as Pb, As, Hg, Cd, V were very low. However, a high amount of As was found in batches 5 and 6 (784 mg/kg and 561 mg/kg), which might have been contaminated during preparation or storage. Concentrations of Na, K, Ca, and Fe were more higher than other elements in the SO, especially in batches 1, 6, 7, and 8. Unexpectedly, Cd and Ni were not detected in batches 1–4, 14, and 15, 18, respectively.

**Table 4:** Contents of 14 types elements in 18 batchs of suet oil (n = 3).

Origins	Batches	Content (mean ± SD, mg/kg)													
		Pb	As	Cd	Hg	Cu	Na	K	Ca	Ti	V	Cr	Mn	Fe	Ni
**Anhui**	1	0.0015 ± 0.0008	0.013 ± 0.005	nd	0.004 ± 0.001	0.82 ± 0.01	817 ± 4	584 ± 5	80 ± 4	21 ± 1	0.274 ± 0.007	9.81 ± 0.03	2.03 ± 0.03	224 ± 5	2.45 ± 0.06
	2	0.18 ± 0.01	0.093 ± 0.006	nd	0.0040 ± 0.0003	1.11 ± 0.09	386 ± 5	248 ± 6	29 ± 1	7.8 ± 0.1	0.14 ± 0.01	8.94 ± 0.04	1.03 ± 0.02	148 ± 6	1.02 ± 0.05
	3	0.20 ± 0.01	0.035 ± 0.007	nd	0.0030 ± 0.0001	1.45 ± 0.02	411 ± 6	314 ± 8	34 ± 4	8.07 ± 0.04	0.15 ± 0.01	8.0 ± 0.1	1.16 ± 0.01	145 ± 3	1.02 ± 0.01
	4	0.11 ± 0.01	0.026 ± 0.004	nd	0.0020 ± 0.0001	0.43 ± 0.01	323 ± 3	206 ± 6	24 ± 2	5.60 ± 0.06	0.113 ± 0.006	7.90 ± 0.05	0.816 ± 0.008	120 ± 5	0.94 ± 0.01
	5	0.70 ± 0.03	784 ± 8	0.012 ± 0.001	0.0002 ± 0.0001	0.276 ± 0.004	360 ± 4	214 ± 5	26 ± 3	8.79 ± 0.07	0.150 ± 0.004	7.9 ± 0.1	1.02 ± 0.01	179 ± 7	0.593 ± 0.008
	6	0.13 ± 0.01	561 ± 11	0.0010 ± 0.0003	0.0015 ± 0.0001	0.239 ± 0.008	632 ± 4	444 ± 9	59 ± 4	19.4 ± 0.1	0.172 ± 0.003	4.07 ± 0.05	1.88 ± 0.02	181 ± 4	3.79 ± 0.07
**Qinghai**	7	1.4 ± 0.1	0.34 ± 0.02	0.030 ± 0.006	0.025 ± 0.001	1.34 ± 0.02	580 ± 7	392 ± 7	50 ± 2	11.61 ± 0.01	0.194 ± 0.002	7.72 ± 0.03	1.36 ± 0.02	157 ± 4	1.42 ± 0.02
	8	0.8 ± 0.1	0.26 ± 0.02	0.016 ± 0.002	0.015 ± 0.001	0.90 ± 0.02	703 ± 8	488 ± 7	59 ± 3	18.57 ± 0.08	0.162 ± 0.004	4.00 ± 0.06	1.68 ± 0.02	188 ± 2	2.38 ± 0.02
	9	1.3 ± 0.1	0.31 ± 0.02	0.020 ± 0.003	0.020 ± 0.001	1.21 ± 0.01	291 ± 5	147 ± 5	26 ± 1	5.76 ± 0.08	0.084 ± 0.005	4.356 ± 0.002	0.91 ± 0.02	93.1 ± 0.2	0.59 ± 0.03
	10	1.4 ± 0.1	0.30 ± 0.02	0.027 ± 0.003	0.018 ± 0.001	1.49 ± 0.02	320 ± 5	159 ± 5	29 ± 3	4.82 ± 0.07	0.224 ± 0.009	6.570 ± 0.002	0.91 ± 0.01	151 ± 3	2.52 ± 0.02
	11	1.2 ± 0.1	0.273 ± 0.008	0.021 ± 0.001	0.0165 ± 0.0004	1.606 ± 0.006	426 ± 5	253 ± 6	41 ± 3	8.2 ± 0.1	0.142 ± 0.001	7.074 ± 0.006	1.04 ± 0.01	126 ± 2	0.99 ± 0.01
**Jiangsu**	12	0.9 ± 0.1	0.104 ± 0.009	0.013 ± 0.001	0.013 ± 0.001	0.844 ± 0.006	296 ± 8	170 ± 6	27 ± 2	6.17 ± 0.04	0.1005 ± 0.0008	7.12 ± 0.04	0.79 ± 0.01	90.9 ± 0.8	2.74 ± 0.03
	13	0.8 ± 0.1	0.04 ± 0.01	0.003 ± 0.001	0.0095 ± 0.0002	0.589 ± 0.009	306 ± 6	170 ± 6	22 ± 1	4.95 ± 0.04	0.099 ± 0.008	7.158 ± 0.013	0.72 ± 0.05	89 ± 2	0.86 ± 0.04
	14	1.2 ± 0.1	0.226 ± 0.006	nd	0.023 ± 0.002	0.90 ± 0.01	246 ± 6	127 ± 7	23 ± 1	4.16 ± 0.03	0.095 ± 0.002	6.08 ± 0.04	0.73 ± 0.05	83 ± 1	0.52 ± 0.02
	15	0.8 ± 0.1	0.21 ± 0.02	0.0055 ± 0.0003	0.0065 ± 0.0002	0.586 ± 0.007	196 ± 5	94 ± 2	18 ± 2	4.55 ± 0.04	0.079 ± 0.003	5.15 ± 0.03	0.62 ± 0.06	79 ± 2	nd
	16	0.7 ± 0.1	0.212 ± 0.008	0.0030 ± 0.0008	0.0090 ± 0.0008	0.53 ± 0.01	264 ± 5	122 ± 3	22 ± 2	3.81 ± 0.02	0.092 ± 0.002	5.96 ± 0.04	0.62 ± 0.01	83.8 ± 0.5	0.134 ± 0.002
	17	0.70 ± 0.07	0.12 ± 0.01	0.0030 ± 0.0008	0.0085 ± 0.0003	0.756 ± 0.007	301 ± 6	141 ± 2	28 ± 2	4.10 ± 0.04	0.094 ± 0.004	5.98 ± 0.06	0.753 ± 0.006	85.8 ± 0.4	1.22 ± 0.01
	18	0.91 ± 0.08	0.041 ± 0.006	0.006 ± 0.001	0.0095 ± 0.0004	0.620 ± 0.004	294 ± 4	141 ± 3	22 ± 2	4.0 ± 0.2	0.086 ± 0.002	5.92 ± 0.02	0.61 ± 0.01	71.6 ± 0.8	nd

nd, not detected (concentration below the LOD).

### 2.3. PCA of the SO Samples

To evaluate the variation of SO, PCA was performed on the basis of the contents of 14 tested elements from SO. The first three principal components (PC1, PC2, and PC3) with >84.6% of the whole variance were extracted for analysis. Among them, PC1 accounted for 51.49% of total variance, whereas PC2 and PC3 for 22.57% and 10.58%, respectively. The remaining principal components were discarded due to their minor effects on the model. The correlation matrix of PCA analysis for 14 elements is shown in Table S3. The total variance for PCA and the components loading matrix is shown in Table 5 and Table 6. According to their loadings, PC1 had good correlation with the 14 elements. The above results suggested that most of the elements might contribute to the classification of the samples.

**Table 5:** The total variance explained for PCA of 14 elements in 18 batches of SO.

Component	Initial Eigenvalues			Extraction Sums of Squared Loadings		
	Total	% of Variance	Cumulative %	Total	% of Variance	Cumulative %
1	7.209	51.491	51.491	7.209	51.491	51.491
2	3.160	22.572	74.062	3.160	22.572	74.062
3	1.482	10.586	84.649	1.482	10.586	84.649
4	0.951	6.794	91.443			
5	0.479	3.423	94.866			
6	0.301	2.153	97.019			
7	0.179	1.280	98.299			
8	0.134	0.960	99.259			
9	0.059	0.418	99.677			
10	0.019	0.133	99.810			
11	0.014	0.102	99.913			
12	0.008	0.060	99.973			
13	0.003	0.018	99.991			
14	0.001	0.009	100.000			

Extraction Method: Principal Component Analysis.

**Table 6:** The component matrix of PCA analysis for 14 elements in 18 batches of SO.

Elements	Component		
	1	2	3
Pb	−0.440	0.825	0.251
As	0.294	−0.400	0.563
Cd	0.032	0.869	0.205
Hg	−0.232	0.902	0.108
Cu	0.018	0.776	−0.469
Na	0.966	0.112	0.001
K	0.970	0.057	−0.042
Ca	0.955	0.152	−0.005
Ti	0.965	−0.012	0.166
V	0.851	0.253	−0.220
Cr	0.226	−0.159	−0.825
Mn	0.982	0.055	0.089
Fe	0.939	0.023	−0.039
Ni	0.732	0.132	0.244

Extraction Method: Principal Component Analysis.

**Figure 3:**
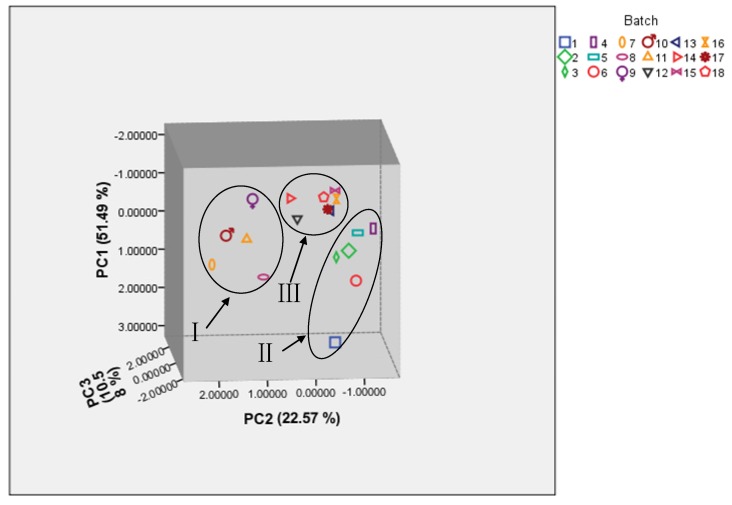
The 3 D scatter plots obtained from PCA of 18 batches SO samples.

In order to further distinguish the diversity of SO samples from different origins, the scatter plot of the study was plotted. We observed that eighteen sample dots were successfully classified into groups I, group II, and group III corresponding to Qinghai, Anhui and Jiangsu (Figure 3). Interestingly, dots in groups II and III were relatively nearer to each other, indicating a closer relationship among six batches from Anhui and seven batches from Jiangsu. However, dots in group I were relatively scattered, suggesting the diversification of the five Qinghai batches. This could be explained by several reasons: firstly, the land area of Qinghai Province is 722,300 square kilometers, larger than Anhui (139,600) and Jiangsu (106,700 square kilometers), which creates advantageous conditions for the diversity of the samples. Secondly, the climate and environment in Qinghai, Anhui and Jiangsu have great differences, which affects the differences in elemental metabolism in *Ovis aries Linnaeus* or *Capra hircus Linnaeus*. Thirdly, Anhui Province and Jiangsu Province are neighboring to each other in geographic location, which is the main reason for the closer results of the samples from the two origins.

## 3. Experimental

### 3.1. General Information

The analytical method used in this study is based on an ultrawave single reaction chamber microwave digestion system (Ultrawave) and inductively coupled plasma-mass spectrometry (ICP-MS), and consists of the following steps: (1) optimization of ultrawave digestion by Box-Behnken design (BBD); (2) measurement of 14 elements in 18 batches of SO by ICP-MS; (3) differentiation of 18 batches of SO by principal component analysis.

### 3.2. Instrumentation

An inductively coupled plasma quadrupole mass spectrometer (Agilent 7700, Agilent, Santa Clara, CA, USA) coupled with an automatic sampler (ASX-500, Agilent) was used in all experiments. The ultrawave single reaction chamber microwave digestion system (Milestone, Milan, Italy) including a 1 L stainless steel reaction chamber and vials and a rack was applied for all SO sample digestions (Table 1 and TableS1).

### 3.3. Reagents and Analytical Solutions

Working standard solutions of 14 elements (Pb, As, Hg, Cd, Fe, Cu, Mn, Ti, Ni, V, Sr, Na, Ka and Ca) were prepared by diluting proper amounts of their inorganic standard solutions (1,000 mg L^−1^ each, Kanto Chemical Industries, Ltd., Tokyo, Japan). Nitric acid and hydrogen peroxide were of ultrapure grade (Kanto Chemical). Pure water (resistivity 18 MΩ cm) was prepared by a Milli-Q water purification system (Millipore Corp., Bedford, MA, USA) and used throughout the experiments.

### 3.4. Samples

In total, 18 batches SO samples were purchased in the Qinghai, Jiangsu and Anhui provinces of China from January 2013 to July 2013, respectively (Figure 1). SO samples of Qinghai were derived from *Capra hircus Linnaeus*, and the samples of Jiangsu and Anhui were obtained from *Ovis aries Linnaeus*. In order to prevent oxidation, all SO samples were stored in a dark environment and kept below 4 °C in a refrigerator.

### 3.5. Sample Preparation Procedure

The SO samples (100 mg) were weighted accurately and placed into vials respectively, and then HNO_3_ (69%, 1.5 mL) and H_2_O_2_ (30%, 0.5 mL) were added. Teflon caps on the glass vials were kept loose to ensure pressure equalization. Sample rack configurations including a 15 position rack for microsamples was used. The temperature and pressure of the ultrawave digestion were maintained at optimal conditions. Sequentially, the digestion procedure was divided into the following five steps: (1) sample rack was lowered automatically into microwave chamber; (2) chamber clamp is secured by the operator. Interlocks prevent operation without this clamp in place; (3) chamber is pre-pressurized with an inert gas to prevent sample boiling. Cross contamination is eliminated; (4) m icrowave energy is applied. All samples are under same temperature and pressure conditions; (5) very fast cooling step due to water cooling of chamber. Chamber is vented and acid vapors extracted; (6) clamp is released and the sample rack automatically rises from chamber. Figure 1 shows how a digestion was performed in the Ultrawave apparatus. After cooling the vials, the solution in the vessel was transferred and made up to volume in a 25 mL volumetric flask. A blank was also prepared in the same manner. 

### 3.6. PCA for SO Samples

PCA was performed to analyze these samples using the SPSS 16.0 software package (SPSS, Chicago, IL, USA). In this study, the contents of the 14 elements which were analyzed from the 18 batches SO samples composed a data matrix with 18 rows and 14 columns, which was used for PCA analysis after normalization. The first three principal components (PCs) were extracted, and the scatter plots were obtained by plotting the scores of PC1 *versus* PC2 and PC3. 

## 4. Conclusions

In this paper, 18 batches of SO samples were analyzed by Ultrawave digestion/ICP-MS with high efficiency, high throughput, lower labor and high accuracy. In addition, the combination of the concentration of multielements with PCA was also a reliable means for the detection and differentiation of trace constituents, which could be used to provide supportive information on inorganic elements contents for evaluating the quality of SO and its products. In summary, the present approach is effective, reliable and can be applied for evaluating the safety and nutritional function of SO and other related products with undefined quality control standards.

## Supplementary Materials

Supplementary materials can be accessed at: http://www.mdpi.com/1420-3049/19/4/4452/s1.
