# Supplementary material for: Multielemental Composition of Suet Oil Based on Quantification by Ultrawave/ICP-MS Coupled with Chemometric Analysis
Source: Molecules. 2014 Apr 10;19(4):4452–65. doi: 10.3390/molecules19044452 (PMC6271827; doi:10.3390/molecules19044452)
Supplement: Supplementary file 1 [file molecules-19-04452-s001.pdf]

Supplementary File

Table S1. Operating conditions and parameters for ICP-MS and ultrawave single reaction chamber microwave digestion system.

	**parameters**
Rf frequency	27.12 MHz
Incident Rf power	1.5 KW
Reflected power	<2 W
Outer gas flow rate	15 L min^{-1}
Intermediate gas flow rate	1.0 L min^{-1}
Carrier gas flow rate	0.9 L min^{-1} for conical nebulizer
Pressure of Argon	700 KPa
Reaction gas pressure (Helium)	40 KPa
Circulating water	Temperature 20 ℃<br>Pressure 230–400 KPa
Exhaust volume	5–7 m^3/min
Compensation gas flow	0.25 L/min
Premix chamber temperature	2 ℃
Integration time	1 second: 75 As^+, 2 seconds: 207Hg^+, 52Cd^+,<br>0.3 seconds: 23Na^+, 26Mg^+,39K^+, 44Ca^+, 52Cr^+, 55Mn^+<br>56Fe^+, 60Ni^+, 63Cu^+, 111Cd^+, 208Pb^+
Peristaltic pump speed	0.1 rps
Measured m/z	23Na^+, 26Mg^+,39K^+, 44Ca^+, 52Cr^+, 55Mn^+ 56Fe^+,<br>60Ni^+, 63Cu^+, 111Cd^+, 208Pb^+, 75 As^+, 207Hg^+, 52Cd^+
Sampling conditions	
Sampling depth	8 mm
Data acquisition	
Scanning mode	E-Scan
Integrated mass window	50%
Data points	20 points/peak
Dwell time	10 ms/point
Integration	5 times
Repetition	5 times

Table S2. Box-Behnken experimental design with there independent variables.

NO.	Digestion Time/min	Digestion Temperature/℃	Digestion Pressure/bar	Recovery/%													
				**Pb**	**As**	**Hg**	**Cd**	**Fe**	**Cu**	**Mn**	**Ti**	**Ni**	**V**	**Cr**	**Na**	**K**	**Ca**
1	50.00	240.00	100.00	84.5	88.4	86.4	87.4	89.6	90.5	96.6	99.5	97.6	96.5	86.2	79.5	74.2	79.5
2	50.00	180.00	100.00	81.4	84.4	85.5	83.5	80.5	86.6	85.3	82.4	91.0	94.3	82.4	72.2	71.0	72.4
3	35.00	210.00	70.00	83.5	80.5	82.5	80.5	78.4	83.5	87.5	88.6	89.4	87.5	86.6	77.2	74.7	78.6
4	35.00	260.45	70.00	86.4	87.6	83.6	81.6	77.5	86.6	84.6	82.1	84.3	85.6	82.1	78.6	69.8	72.1
5	35.00	159.55	70.00	62.3	75.4	73.4	71.4	68.5	69.4	67.4	71.3	73.1	76.4	81.3	66.4	71.2	70.4
6	20.00	180.00	100.00	71.0	79.2	83.7	80.7	82.1	77.6	80.4	74.2	76.4	75.6	74.2	54.7	46.8	74.3
7	20.00	240.00	40.00	63.1	67.9	60.7	58.7	63.3	68.7	69.7	64.2	69.9	69.7	63.4	49.7	48.7	47.2
8	35.00	210.00	70.00	87.5	86.6	84.6	81.6	78.9	82.6	83.1	87.6	89.6	88.6	87.6	70.6	72.6	77.2
9	9.77	210.00	70.00	58.2	60.2	65.3	66.3	70.3	65.4	65.7	64.5	52.4	57.4	54.7	36.4	45.5	54.3
10	35.00	210.00	120.45	90.4	91.4	93.5	91.5	89.5	88.1	94.1	92.5	97.5	91.1	95.5	72.1	71.1	72.5
11	35.00	210.00	70.00	83.4	80.3	79.4	77.4	74.8	73.6	79.5	79.6	82.5	86.1	89.4	69.5	67.1	79.6
12	20.00	180.00	40.00	60.2	56.7	53.9	50.9	61.1	53.9	48.3	67.4	60.3	57.9	67.4	50.2	48.9	57.4
13	50.00	180.00	40.00	54.2	60.4	52.3	50.3	57.2	58.3	62.1	68.2	63.5	58.3	58.2	38.3	41.3	58.2
14	50.00	240.00	40.00	60.4	60.9	57.2	55.2	57.6	62.2	66.2	72.4	74.5	68.2	72.4	54.2	50.5	62.4
15	35.00	210.00	19.55	57.4	47.5	48.4	53.1	51.2	56.7	65.7	63.7	67.1	64.8	63.7	43.4	36.3	53.7
16	60.23	210.00	70.00	88.4	90.9	86.9	87.7	85.7	83.9	87.6	85.1	86.3	88.9	85.1	48.9	38.9	55.1
17	35.00	210.00	70.00	88.2	82.7	84.4	85.45	83.7	87.4	87.8	84.6	89.4	88.4	84.6	68.4	64.7	71.6
18	35.00	210.00	70.00	87.6	85.6	86.7	87.1	84.6	84.5	90.3	92.4	94.3	87.3	92.4	77.3	75.9	82.4
19	20.00	240.00	100.00	85.3	83.4	81.5	79.3	79.8	81.5	78.4	83.4	77.3	68.5	77.5	78.5	75.7	81.5
20	35.00	210.00	70.00	83.7	85.2	86.4	84.2	85.8	87.4	86.4	88.4	90.3	87.4	80.4	77.4	72.4	80.4

Table S3. The correlation matrix of PCA analysis for 14 elements in 18 batches of SO.

		**Pb**	**As**	**Cd**	**Hg**	**Cu**	**Na**	**K**	**Ca**	**Ti**	**V**	**Cr**	**Mn**	**Fe**	**Ni**
Correlation	Pb	1.000	−0.220	0.781	0.891	0.462	−0.334	−0.404	−0.300	−0.402	−0.171	−0.349	−0.382	−0.376	−0.212
	As	−0.220	1.000	−0.053	−0.416	−0.525	0.157	0.163	0.114	0.303	0.154	−0.079	0.284	0.408	0.216
	Cd	0.781	−0.053	1.000	0.698	0.558	0.094	0.034	0.095	0.011	0.268	−0.169	0.054	0.132	0.160
	Hg	0.891	−0.416	0.698	1.000	0.578	−0.093	−0.149	−0.050	−0.189	−0.030	−0.273	−0.149	−0.241	−0.051
	Cu	0.462	−0.525	0.558	0.578	1.000	0.072	0.072	0.112	−0.080	0.278	0.187	0.045	0.075	0.033
	Na	−0.334	0.157	0.094	−0.093	0.072	1.000	0.991	0.980	0.965	0.791	0.158	0.968	0.871	0.649
	K	−0.404	0.163	0.034	−0.149	0.072	0.991	1.000	0.965	0.964	0.777	0.196	0.972	0.879	0.640
	Ca	−0.300	0.114	0.095	−0.050	0.112	0.980	0.965	1.000	0.953	0.805	0.139	0.963	0.831	0.684
	Ti	−0.402	0.303	0.011	−0.189	−0.080	0.965	0.964	0.953	1.000	0.721	0.034	0.981	0.865	0.690
	V	−0.171	0.154	0.268	−0.030	0.278	0.791	0.777	0.805	0.721	1.000	0.418	0.791	0.885	0.628
	Cr	−0.349	−0.079	−0.169	−0.273	0.187	0.158	0.196	0.139	0.034	0.418	1.000	0.095	0.315	−0.061
	Mn	−0.382	0.284	0.054	−0.149	0.045	0.968	0.972	0.963	0.981	0.791	0.095	1.000	0.900	0.717
	Fe	−0.376	0.408	0.132	−0.241	0.075	0.871	0.879	0.831	0.865	0.885	0.315	0.900	1.000	0.617
	Ni	−0.212	0.216	0.160	−0.051	0.033	0.649	0.640	0.684	0.690	0.628	−0.061	0.717	0.617	1.000
